# Future implications of ChatGPT in pharmaceutical industry: drug discovery and development

**DOI:** 10.3389/fphar.2023.1194216

**Published:** 2023-07-17

**Authors:** Ailin Zhao, Yijun Wu

**Affiliations:** ^1^ Department of Hematology, West China Hospital, Sichuan University, Chengdu, Sichuan, China; ^2^ Cancer Center, West China Hospital, Sichuan University, Chengdu, Sichuan, China

**Keywords:** ChatGPT, academic writing, clinical drug, drug discovsry, artificial intelligence

## Introduction

The process of developing an innovative drug is convoluted. Although the COVID-19 pandemic has accelerated the development of COVID-19-related vaccines and drugs, it usually takes several years or decades to gain approval for clinical application from initial preclinical experiments. Consequently, it is of an urgent need to shorten the development period based on scientific principles and strict supervision, in order to provide patients with improved treatment response and prognosis. The recently proposed ChatGPT launched in November 2022 is a promising artificial intelligence learning model that has sparked great interest in academic writing (OpenAI: ChatGPT, 2022). After giving input texts, it can rapidly generate intelligent information like human based on a large amount of data with multiple languages, which relies on a neural nwtwork architecture to deal with natural language (Sallam, 2023). By combining ChatGPT’s efficient natural language processing ability with innovative drug development, it is expected to bring about unprecedented ideas and breakthroughs, ultimately accelerating the development of innovative drugs.

## Discussion

The discovery of hit/lead compounds is a critical step in the drug development process. In this regard, the recently proposed ChatGPT model shows great promise in rapidly recognizing and validating novel drug targets, as well as designing and improving hit compounds while screening the compound database. First, based on the powerful language processing and analyzing capability, ChatGPT can explore published literatures and patent databases to identify potential hit compounds. After comprehensive analysis of the vast literature, ChatGPT can recognize disease-specific agents, compounds, genes and other relevant information. ChatGPT can bridge the communication gap among researchers in different specialties, and provide essential clues for drug research and development. ChatGPT can design compounds with new structures based on their physical and chemical features, thereby assisting researchers in achieving more effective drug discovery. Furthermore, ChatGPT can predict the pharmacokinetic (PK), pharmacodynamic (PD), and toxicity features of a particular compound, providing vital information for drug development. An example of ChatGPT’s application is in protein drug design (Heck, 2023). The conversation about amino acid sequences in the protein database is input into the program. The conversation involves the structural domain, specific function, and configuration of proteins. ChatGPT analyzes, studies, and codes the information above, leading to generation of new protein sequences and structures that ensure precision and synthesizability, thus leading the protein design evolution.

Gaurav et al. has investigated the performance of ChatGPT in drug discovery, revealing promising results (Thakur and Sharma, 2023). In the field of computational chemistry, ChatGPT has demonstrated the ablility to accurately obtain compound multiplicity, which has accelerated the generation of input files for Gaussuan software and the identification of protein data bank files. However, for more complex issues, such as the retrieval of FASTA sequences and ADMET properties, ChatGPT still requires further development (Thakur and Sharma, 2023). Overall, ChatGPT offers a cost-effective approach to handle massive data and generate new knowledge, which can assist researchers with decision making and expedite drug discovery. However, it is important to note that the validation of ChatGPT’s prediction through preclinical experiments and clinical trials is essential and cannot be replaced by the use of ChatGPT alone.

Different from the relatively standardized preclinical PK, PD, and toxicologic studies, clinical trials are complex processes involving various stages. Due to the difference in participants’ biochemical features, the introduction of artificial intelligence to clinical trials poses a challenge. However, after solving the bottleneck problem, ChatGPT’s powerful network can significantly reduce the length of clinical trials to a matter of months or years (Thakur and Sharma, 2023). Before the emergence of ChatGPT, disease-related data were often isolated and difficult to share globally, requireding extra cooperation to promote clinical trial efficiency. However, ChatGPT is distinct from traditional clinical decision making. It can effectively integrate existing disease and clinical trial data, creating data sets with standardized structures. ChatGPT’s intellectual differentiation, analysis, and data transmission can minimize drug development failures and socioeconomic burdens. Moreover, ChatGPT can extract insights from previous and current clinical trials, improving future trials. Consider the potential impact of ChatGPTon failed clinical trials if it had emerged earlier.

The COVID-19 pandemic has exposed various limitations in drug development, highlighting the need to accelerate the process (Asselah et al., 2021). Traditional methods for identifying disease targets and potential therapeutic chemicals are often inefficient, requiring manual selection and validation or omics analysis by a bioinformatician (Yang et al., 2022). Additionally, the vast amount of literature available makes it difficult to consult and extract meaningful information. The emergence of ChatGPT presents a valuable evolution that can overcome these limitations. In detail, a variety of practical scenarios for ChatGPT can be further explored in drug discovery and development: 1) integration of a plethora of diseases-related information, such as causal genes or molecules, structures, and characteristics of other effective therapeutic agents, to search for potential new drugs; 2) simulation of drug metabolism and distribution *in vivo* to suggest optimal dosage and regimen, which can enhance efficacy and minimize adverse effects; 3) prediction and explanation of drug-drug or drug-protein interactions, which are crucial in target selection and drug design (Juhi et al., 2023); 4) optimization of the design and execution of clinical trials to improve the success rate and reliability of drug development, thus expediting the introduction of new drugs into the clinic; 5) provision of personalized medicine-based strategies, which entail the analysis of individual patient’s genetic information, human genetics, and drug characteristics; 6) contribution to pharmacoeconomic evaluations of new drugs that can aid in guiding national health insurance policies.

In spite of the great potential of ChatGPT in drug discovery and development, there are several limitations that need to be addressed. First, as with other machine learning-based algorithms or models, ChatGPT is often unexplainable, and the source of its predictive outcomes cannot be traced (Azodi et al., 2020; Musolf et al., 2022; Petch et al., 2022). This lack of transparency makes it difficult to provide evidence for the suggestions it generates. However, researchers are working on ways to make ChatGPT more explainable and practical in the future. Second, the current version of ChatGPT has not been trained on sufficient data (OpenAI: ChatGPT, 2022), so its predictability needs further optimization. Third, there are always unpredictable events in the real world, which cannot be entierly avoided, even with the use of ChatGPT. Therefore, more reliable input data are necessary to improve its accuracy. Besides, although ChatGPT can predict outcomes and simulate drug-related processes, it cannot perform experimental validations. Finally, and perhaps most important, it is still difficult for ChatGPT to completely achieve critical thinking like humans (Davies, 2023). However, we nowadays do not know what will happen when artificial intelligence could think independently. In short, ChatGPT will be improved in the future and its application in pharmacology and medicine will be further developed and innovated to accelerate drug development and benefit patients.
